# Delayed recognition of fatal invasive meningococcal disease in adults

**DOI:** 10.1099/jmmcr.0.005027

**Published:** 2016-06-28

**Authors:** Frederick W. Nagel, Ifeoma Ezeoke, Mike Antwi, Paula E. Del Rosso, Marie Dorsinville, Beth M. Isaac, Althea Hayden, Robert S. Hoffman, Scott D. Weingart, Don Weiss

**Affiliations:** ^1^​ Department of Emergency Medicine, North Central Bronx Hospital, Albert Einstein College of Medicine, Bronx, NY, United States; ^2^​ New York City Department of Health and Mental Hygiene, New York, NY, United States; ^3^​ Division of Medical Toxicology, Ronald O. Perelman Department of Emergency Medicine, NYU School of Medicine, NY, USA; ^4^​ Division of Emergency Critical Care, Stony Brook Hospital, Stony Brook, NY, USA

**Keywords:** Meningococcal Infections/complications, Meningococcal Infections/prevention & control, Neisseria meningitidis, New York City/epidemiology, Risk Factors, United States/epidemiol

## Abstract

**Introduction:**

Invasive meningococcal disease can be difficult to detect early in its course when patients may appear well and the severity of their illness is obscured by non-specific complaints.

**Case presentation:**

We report five cases of meningococcal sepsis in adult patients who presented to an emergency department early in the course of their disease, but whose severity of illness was not recognized.

**Conclusion:**

Suspicion of meningococcal sepsis should be heightened in the setting of hypotension, tachycardia, elevated shock index, leukopaenia with left shift, thrombocytopaenia and hypokalaemia, prompting early sepsis care.

## Introduction

Meningococcal disease is particularly devastating as many of its victims are young and otherwise healthy. In New York City, a recent outbreak of invasive meningococcal disease among men who have sex with men resulted in the death of seven of the 22 infected men (Kratz *et al.*, 2015). According to the most recent national data, there were 556 cases in the United States in 2013 and an average of 88 deaths per year from meningococcal disease between 2005 and 2011 (CDC, 2015). Meningococcal disease presents a diagnostic challenge to the astute emergency physician; its early presentation may be non-specific, yielding only subtle clues of septic shock (Rosenstein *et al.*, 2001). In reviewing meningococcal cases in New York City since 2000, we focused on those patients who had been evaluated in an emergency department (ED) early in the course of their illness, but whose severity of illness was unrecognized. The following cases were abstracted from the hospital medical records and the New York City Department of Health and Mental Hygiene investigative reports of patients with invasive meningococcal disease. While cases from all serogroups of *Neisseria meningitidis* were considered, those presented in this series were caused by serogroup C.

## Case report

### Case 1

A woman in her early twenties with a history of asthma was ambulatory on arrival to an academic ED in 2008 complaining of sore throat for 3 days that was associated with weakness, fever, productive cough and back pressure. At triage her oral temperature was 37.8 °C (100.1 °F), heart rate was 136 beats min^−1^ and blood pressure was 120/60 mmHg. The shock index (heart rate divided by systolic blood pressure) was 1.13 (normal ≤0.7, Rady *et al.*, 1992). Physical examination revealed a normal oropharynx and expiratory wheezes. During the course of her visit, a second oral temperature was measured at 39.4 °C (103.0 °F). Chest radiograph showed basilar streaky opacities in one view. No laboratory studies were sent. Vital signs were repeated 5 h after arrival and showed a heart rate of 112 beats min^−1^ and a blood pressure of 100/60 mmHg (shock index 1.12). The patient was discharged with a diagnosis of upper respiratory infection of likely viral aetiology.

Thirteen hours later she returned to the ED by ambulance complaining of pain and red spots on her hands and feet. Vital signs revealed an oral temperature of 37.2 °C (98.9 °F), heart rate of 120 beats min^−1^ and a blood pressure of 92/60 mmHg (shock index 1.3). The patient was described as tachypnoeic, with a respiratory rate of 30 breaths min^−1^ and hypoxic with an oxygen saturation of 90 % on room air. She also complained of a mild headache, but had no nuchal rigidity or neurological abnormality on examination. Purpura was noted on her extremities and trunk. Although she received appropriate intravenous antibiotics 2 h after triage, her condition deteriorated and she died in the intensive car unit (ICU) 6 h after her arrival. *N. meningitidis* was isolated from her blood cultures and autopsy showed adrenal haemorrhage, consistent with meningococcaemia.

### Case 2

A man in his mid-twenties with a history of human immunodeficiency virus (HIV) infection on anti-retroviral treatment and recent crystal methamphetamine use arrived ambulatory to an academic ED in 2013. At triage, the patient reported flu-like symptoms, including fever, flank pain, sore throat and myalgias for 1 week, and diarrhoea for the past day; his presenting complaint was recorded as 'I think I have meningitis or flu'. The patient reported a history of meningitis which was described as 'viral'. His oral temperature was 38.7 °C (101.7 °F), heart rate was 135 beats min^−1^, blood pressure was 103/64 mmHg and shock index was 1.31. The patient’s CD4 count was unknown at the time of his visit, but was in fact greater than 400 cells mm^-3^.

On physical examination, the patient was in no apparent distress and had a normal gait, no nuchal regidity, pharyngeal erythema or reproducibleback pain. The patient was given a 2 litre intravenousbolus of normal saline and 975 mg of acetominophen orally. Three hour later the patient's vital signs continued to be abnormal (heart rate 124 beats min-1, blood pressure 94/56 mmHg). Ibuprofen 600 mg was given orally and a continuous infusion of normal saline was ordered. Blood tests revealed a total white blood cell count of 3000 mm^-3 ^with 84 % neutrophils and a platelet count of 139 000 mm^-3^. Venous lactate was measured at   2.8 mmol l^−1^, venous pH was 7.38, serum bicarbonate was 25 mmol l^−1^ and anion gap was 10. Serum potassium was measured at 3.0 mmol l^−1^ and was repleted intravenously and orally. Urinalysis, influenza antigen test and chest radiography were normal. The patient was noted to be feeling somewhat improved and observed walking to the bathroom; the patient was signed out to the next shift.

Six hours after triage, the patient’s condition continued to decline with a blood pressure of 82/46 mmHg and heart rate of 121 beats min^−1^. Parenteral antibiotics, corticosteroids and vasopressors were started nearly 9 h after triage when the patient was noted to be dyspnoeic. Despite resuscitative measures, the patient died 10 h after triage. *N. meningitidis* was cultured from the patient’s blood.

### Case 3

A woman in her mid-fifties with a history of non-Hodgkin’s lymphoma in remission and osteoporosis was brought by ambulance to an academic ED in 2010 complaining of extremity pain from a fall 1 h earlier. In addition, the patient complained of fever and a sore throat for the past day and had taken acetaminophen after the fall. At triage the patient’s oral temperature was 38.4 °C (101.1 °F), heart rate was 115 beats min^−1^ and blood pressure was 101/64 mmHg (shock index 1.14).

The medical record described a mechanical trip and fall, and the patient complained of shoulder and hip pain; the physical examination was consistent with contusions sustained from the earlier fall. No neck stiffness was present and the pharynx was noted to be erythematous. Plain radiographs of the extremities showed no fracture, a throat culture was performed and 975 mg of acetaminophen was given; no other laboratory analyses were performed. Three hours after triage the patient’s temperature had normalized to 37.1 °C (98.8 °F) and heart rate had decreased to 101 beats min^−1^; no repeat blood pressure was recorded. The patient was discharged with instructions to follow up promptly.

Twenty-four hours later the patient was brought back to the ED by ambulance with confusion, tachycardia (133 beats min^−1^) and hypotension (84/56 mmHg, shock index 1.58); a petechial rash was noted on her extremities. Despite fluid resuscitation, parenteral antibiotics and corticosteroids, the patient died 11 h after her second ED arrival. Cultures of cerebrospinal fluid and blood grew *N. meningitidis*.

### Case 4

A woman in her early forties with a history of a herniated disc arrived ambulatory to a community ED in 2013 complaining of sore throat and fever which had started the night before. She also complained of difficulty walking, which was attributed to chronic spine disease, with numbness to the extremities. At triage, she had an oral temperature of 37.8 °C (100.1 °F), heart rate of 101 beats min^−1^ and blood pressure of 100/66 mmHg, which yields a shock index of 1.01. To her physician, the patient reported ear numbness as well as lateral foot numbness, which was characterized as chronic.

The physical examination was unremarkable, without abnormality in the pharynx, neck, spine or skin. The neurological examination was normal and the patient was described as ambulating without difficulty. A rectal temperature revealed a fever (39.5 °C/103.1 °F). Laboratory studies were notable for a total white blood cell count of 4000 mm^-3 ^with 88 % neutrophils, and platelet count of 155 000 mm^-3^. Serum potassium was measured at 3.3 mmol l^−1^. The patient was given 2 l of intravenous normal saline, acetaminophen, diazepam 5 mg orally and morphine 6 mg intravenously. Repeat vital signs showed a heart rate of 98 beats min^−1^ and a blood pressure of 112/65 mmHg (shock index 0.88). She was discharged around 4 h after arrival with a diagnosis of pharyngitis and viral syndrome and was prescribed ibuprofen and a short course of diazepam and oxycodone with acetaminophen.

Six hours later emergency medical services discovered the patient on her bedroom floor in cardiac arrest. Resuscitative measures were unsuccessful. Cerebrospinal fluid collected at autopsy confirmed the presence of *N. meningitidis*.

### Case 5

In 2008, a woman in her early forties with no pertinent medical history was brought by ambulance to an academic ED complaining at triage of dizziness and flu-like symptoms for 2 weeks. The physician’s note focused on a complaint of acute left lower quadrant abdominal pain which had begun 2 h earlier. The abdominal pain was associated with flank pain, subjective fever, nausea and generalized weakness. The patient also reported 2 weeks of fever, sore throat, dry cough and nasal congestion. At triage her oral temperature was 36.4 °C (97.6 °F), heart rate 97 beats min^−1^, blood pressure 126/76 mmHg and shock index 0.77.

While she was described as being in mild distress, her physical examination revealed a supple neck, normal pharynx and moderate low abdominal tenderness. No rash was noted on the skin. A differential including diverticulitis was entertained; the patient was prepared for a computed tomography (CT) scan of the abdomen, and she was given 2 l of normal saline and 10 mg of intravenous metoclopramide.

Laboratory studies revealed a total white blood cell count of 1800 mm^-3 ^with 78 % neutrophils, and a platelet count of 144 000 mm^-3^. Serum bicarbonate was 23 mmol l^−1^, serum potassium was 3.4 mmol l^−1^ and anion gap was 12. Two hours after triage, the patient’s rectal temperature was 40.1 °C (104.2 °F). The patient began feeling worse, having chills, and more intravenous fluids were given with 975 mg of oral acetaminophen. Approximately 4 h after triage the patient’s abdominal pain worsened and she developed a rash. The patient was treated with 4 mg of ondansetron and 4 mg of morphine. Repeat vital signs revealed a blood pressure of 98/55 mmHg and heart rate of 106 beats min^−1^ (shock index 0.92). Parenteral antibiotics were started 5 h after triage. Serum lactate was measured at 6.4 mmol l^−1^. The patient underwent a CT scan which showed generalized permeability of tissues suggestive of shock. Her rash developed into a full-body petechial eruption and the patient was placed in isolation pending transfer to the ICU. Her condition continued to deteriorate and she died in the ICU less than 17 h after her ED arrival. Blood cultures confirmed *N. meningitidis* bacteraemia.

## Discussion

These cases presented diagnostic challenges for providers given the diverse and often non-specific nature of the patients’ complaints. However, there were signs that could have guided more aggressive therapy. Consistently, patients were found to be tachycardic and hypotensive, often at triage, with an abnormal shock index (Table 1). The shock index is gaining interest as a tool to recognize early sepsis, alerting providers to subtle abnormalities in heart rate and blood pressure (Rady *et al.*, 1994; Berger *et al.*, 2013; Tseng & Nugent, 2015). All patients in this series met criteria for sepsis which would have prompted further diagnostic testing in institutions with sepsis screening protocols, such as New York’s STOP Sepsis Collaborative screening tool (Dellinger *et al.*, 2013; Greater New York Hospital Association, 2013). When laboratory studies were obtained, the white blood cell count was often low and a left shift (increased percentage of neutrophils) was present. A white blood cell count less than 10 000 mm has been associated with poor prognosis in patients with disease meningococcal (Lodder *et al.*, 1996). Platelet counts were frequently borderline low at initial presentation, and often decreased precipitously as illness progressed and disseminated intravascular coagulation developed. Serum potassium, when measured, was often below normal range. In cases not included in this series, serum potassium was as low as 2.6 mmol l^−1^. Hypokalaemia can result from an increase in circulating catecholamines, as might be expected in patients with early sepsis (Brown *et al.*, 1983). This may be clinically useful as providers in these cases were quick to replete potassium even when early sepsis was unrecognized and antibiotic therapy was delayed.

**Table 1.** Summary of vital signs and laboratory results

	Case 1	Case 2	Case 3	Case 4	Case 5	Normal
**Triage evaluation**						
HR (beats min^−1^)	136	135	115	101	97	–
BP (mmHg)	120/60	103/64	101/64	100/66	126/76	–
Shock index	1.13	1.31	1.14	1.01	0.77	≤0.70
**Repeat evaluation**						
HR (beatsmin^−1^)	112	124	101	98	106	–
BP (mmHg)	100/60	94/56	–	112/65	98/55	–
Shock index	1.12	1.32	–	0.88	1.08	≤0.70
**Laboratory Studies**						
WBC (× 10^9^ l^−1^)	–	3.0	–	4.0	1.8	4.0–10
(Neut %)		(84)		(88)	(78)	(40–60)
Platelets (× 10^9^ l^−1^)	–	139	–	155	144	150–350
Potassium (mmol l^−1^)	–	3.0	–	3.3	3.4	3.5–5.0

HR, heart rate; BP, blood pressure; WBC, white blood cell count; Neut, neutrophil.

These cases were also vexing because they violated the emergency medicine truism that numerous unrelated complaints provide reassurance that none is particularly serious (Veysman, 2011; Ofri, 2010). In most cases in this series providers were perhaps distracted by multiple vague complaints. However, meningococcal sepsis can manifest non-specific symptoms in different organ systems: in Case 3, sepsis probably caused the patient’s generalized weakness and consequent fall; in Case 5 the patient’s severe abdominal pain was caused by her septic shock. Among other New York City cases not presented in this series, signs of meningococcal sepsis were attributed to illicit drug use or cardiac tachyarrhythmia. A longer duration of flu-like symptoms may seem inconsistent with severe disease, but a concurrent viral infection is a recognized risk factor for meningococcal infection (Rosenstein *et al.*, 2001). Many patients in this series complained of myalgias or back pain. While seemingly non-specific, myalgias and extremity pain are strongly associated with meningococcaemia in children and adults (Louria *et al.*, 1985; Thompson *et al.*, 2006). From 2010 to June 2013, nearly a half of New York City adult patients with meningococcaemia reported back or extremity pain. Furthermore, despite signs suggestive of systemic illness, the encounters focused on a single, concrete issue, especially if it was the focus of the patient’s triage. This is related to the phenomenon of anchoring, where a physician fixates on a particular feature of a patient’s presentation to the detriment of a more comprehensive investigation (Corskerry, 2002). In addition, the physician’s interpretation that a patient’s presentation is reflective of a chronic, stable process that has been previously worked up can also result in a truncated evaluation (Case 4). Moreover, patients may be considered too young or otherwise healthy to suffer any serious illness, especially when the prevalence of such illness is low (Cases 1 and 2). Some patients in this series were described as well in appearance, walking to the bathroom for example, despite abnormal vital signs or laboratory values; providers may have been misled by a deceptive clinical impression. Lastly, providers may have conflated meningococcal meningitis with meningococcal septicaemia. Only about 50 % of patients with meningococcal disease will develop meningitis (Rosenstein *et al.*, 2001). The absence of typical meningeal signs seemed to provide false reassurance to the physician, perhaps delaying treatment despite systemic illness and even rash (Cases 1 and 5). Meningeal infection represents a separate manifestation of meningococcal disease and is associated with better survival than meningococcal sepsis (van Deuren *et al.*, 2000). Cerebrospinal fluid analysis, if performed, may be inconclusive in patients with meningococcaemia alone (van Deuren *et al.*, 2000).

It remains difficult to distinguish the early stages of bacterial sepsis from more prevalent viral syndromes. However, abnormal vital signs, including the presence of elevated shock index, in patients with an infection should prompt providers to evaluate for sepsis. A careful history and physical examination followed by laboratory studies may reveal leukopaenia, especially with left shift, borderline thrombocytopaenia or hypokalaemia. In patients with this constellation of findings, prompt sepsis care, including empirical antibiotics, is recommended. Rectal thermometry is encouraged to diagnose fevers which may be missed with oral thermometry (Barnett *et al.*, 2011). Careful examination of the skin and mucous membranes should be performed with the objective of identifying haemorrhagic lesions which are present in 28–77 % of patients with meningococcal septicaemia (Fernandez-Frackelton, 2010). For patients with borderline vital signs during their ED evaluation, vital signs should be systematically reassessed prior to discharge.

Further studies are needed to characterize the sensitivity and predictive values of the abnormalities identified in this series, with special attention to the combination of signs that seems most suggestive of underlying illness. Addressing common pitfalls in clinical decision-making may have widespread benefit for patient care. While occult bacteraemia continues to confound even the most attentive and knowledgeable practitioner, there may be early indications for increased scrutiny, early intervention, or observation and reassessment.
